# Expression of IRAK1 in Hepatocellular Carcinoma, Its Clinical Significance, and Docking Characteristics with Selected Natural Compounds

**DOI:** 10.3390/curroncol29110700

**Published:** 2022-11-18

**Authors:** Chaoying Song, Xinyu Gu, Ruifang Li

**Affiliations:** Department of Pharmaceutical Sciences, School of Basic Medical Sciences, Henan University of Science and Technology, Luoyang 471023, China

**Keywords:** IRAK1, hepatocellular carcinoma, TP53 mutation, targeted therapy, IRAK1/NF-κB signaling, natural compound

## Abstract

This study aimed to explore clinical significance of interleukin-1 receptor-associated kinase 1 (IRAK1) in the diagnosis, prognosis, and targeted therapy of hepatocellular carcinoma. A systematic analysis based on the cancer genome atlas (TCGA) indicated that IRAK1 was highly expressed in 18 cancer types (*p* < 0.01) and may be a pan-cancer biomarker. In hepatocellular carcinoma, the alteration rate of IRAK1 was rather high (62.4%), in which mRNA high relative to normal predominated (58.9%). Higher expression was associated with shorter overall survival (*p* < 0.01). IRAK1 expression correlated positively with pathology stage and tumor grade (for the latter there was only a slight trend). Interestingly, it correlated positively with TP53 mutation (*p* < 0.001), suggesting a possible strategy for targeting TP53 via IRAK1. Immunohistochemistry experiments confirmed a higher positive rate of IRAK1 in carcinoma than in para-carcinoma tissues (χ^2^ = 18.006, *p* < 0.001). Higher tumor grade correlated with more strongly positive staining. Molecular docking revealed cryptotanshinone, matrine, and harmine as the best hit compounds with inhibition potential for IRAK1. Our findings suggest that IRAK1 may play biologically predictive roles in hepatocellular carcinoma. The suppression of IRAK1/NF-κB signaling via inhibition of IRAK1 by the hit compounds can be a potential strategy for the targeted therapy.

## 1. Introduction

Liver cancer ranks sixth in the most commonly diagnosed cancers and fourth in the leading causes of cancer death worldwide. The prognosis is poor and the survival time is short. It ranks second for males and sixth for females in the mortality of all cancer types. As for histological subtypes, hepatocellular carcinoma predominates (75–85% of cases) in primary liver cancer [1]. Early diagnosis and treatment are highly important for prolonging the survival of patients with hepatocellular carcinoma. In particular, correlations between histological features and genetic alterations of hepatocellular carcinoma, which are not very clear so far, need to be revealed so as to find useful biomarkers for early diagnosis [2]. Further, for the purpose of targeted therapy, the discovery of therapeutic targets is crucial. Importantly, combination therapies that include the use of natural compounds targeting certain hallmarks are promising for the treatment of hepatocellular carcinoma [3].

Currently, the approved first-line systemic drug sorafenib has limitations due to multiple adverse events, which may cause its permanent discontinuation. The management of adverse events that aims at a longer treatment period and patients’ survival is difficult and requires physicians with good experience [4]. Moreover, upon the failure of first-line treatment, currently available standard second-line agents for hepatocellular carcinoma are scarce. Recent studies described the safety and efficacy profiles of metronomic capecitabine [5], regorafenib [6], cabozantinib [7], and ramucirumab [8], and revealed their roles as potential second-line treatments for hepatocellular carcinoma. Novel approaches for the targeted therapy of hepatocellular carcinoma, especially novel targets and relative targeted agents, are still urgently needed.

Inflammatory cytokine IL-6 and downstream proteins such as nuclear factor kappa-B (NF-κB), c-Jun N-terminal kinase (JNK), and signal transducer and activator of transcription 3 (STAT3) promote the development of hepatocellular carcinoma, indicating that inflammatory cytokine signals could be possible therapeutic targets [9]. Importantly, interleukin-1 receptor-associated kinase 1 (IRAK1), a member of the IRAK kinase family, plays a key role in the innate immune system through Toll-like receptor (TLR) and interleukin-1 receptor (IL-1R) signaling [10]. It is a key node of the IRAK1/tumor necrosis factor receptor-associated factor 6 (TRAF6)/NF-κB signaling, which is related to inflammation-associated carcinogenesis [10,11]. IRAK1 is overexpressed in most hematologic malignancies [11], non–small cell lung carcinoma [12], and hepatocellular carcinoma [13]. In breast cancer, it is down-regulated after neoadjuvant chemotherapy [14]. The knockdown of IRAK1 suppresses the growth of hepatocellular carcinoma cells [15]. Moreover, the inhibition of IRAK1 makes tumor cells sensitive to radiotherapy [16] and chemotherapy [17]. There are reported synthetic IRAK1 inhibitors designed for therapeutic purposes against tumor or inflammatory diseases [10,11,16].

Until now, the specific clinical significance of IRAK1 expression in hepatocellular carcinoma is not fully revealed, especially its correlation with clinicopathological parameters. Moreover, as the key node of IRAK1/TRAF6/NF-κB signaling, IRAK1 is rarely investigated in terms of screening of natural compounds for potential inhibitors and signaling suppressors to be used in the targeted therapy of hepatocellular carcinoma. Herein, we systematically explored the importance of IRAK1 in the diagnosis, prognosis, and targeted therapy of hepatocellular carcinoma. The cancer genome atlas (TCGA) that integrates molecular characteristics from 33 cancer types [18] was utilized for analysis. Immunohistochemistry experiments were performed to detect IRAK1 expression in liver tissues from clinical specimens. Correlations between IRAK1 expression profiles, clinicopathological parameters, and survival profiles of hepatocellular carcinoma were revealed. Molecular docking with representative natural compounds was carried out to screen potential IRAK1 inhibitors. Further, the possible involvement of the hit compounds in the IRAK1/NF-κB pathway was proposed. This work was undergone to provide potential diagnostic biomarkers and therapeutic targets for hepatocellular carcinoma, and to afford hit natural compounds as potential IRAK1 inhibitors and suppressors of IRAK1-related pathways to be used in targeted therapy.

## 2. Materials and Methods

### 2.1. Pan-Cancer, Gene Alteration and Survival Analyses

TIMER 2.0 (http://timer.comp-genomics.org/, accessed on 25 June 2022) [19] was used for pan-cancer analysis. The ‘Gene_DE’ module was used to explore the differential expression of IRAK1 between tumors and adjacent normal tissues across TCGA cancer types. A comparison was made for each cancer type when normal data were available. The statistical significance was evaluated by Wilcoxon test.

The alterations of the IRAK1 gene in hepatocellular carcinoma were analyzed using cBioPortal (https://www.cbioportal.org/, accessed on 24 March 2022) [20]. The dataset of liver hepatocellular carcinoma (LIHC) from TCGA PanCancer Atlas was selected. The genomic profiles comprised mutation, structural variant, putative copy-number alteration, and mRNA expression *z*-scores relative to normal (calculated using mRNA expression of normal samples as reference data, logRNASeqV2 RSEM, *z*-score threshold ± 2.0) [21]. The samples with complete data were selected as the patient set (348 samples). The onco-print function was used to exhibit the results. Further, the relationships between IRAK1 expression and prognosis of hepatocellular carcinoma were investigated using GEPIA (http://gepia.cancer-pku.cn/, accessed on 22 March 2022) [22]. The survival analysis module was used. The Kaplan–Meier model and Log-rank test were employed for plotting and statistics, respectively. The LIHC dataset was selected. The group cutoff, which is the expression threshold for splitting the high-expression and low-expression groups within the dataset, was set at median. Other analyzing conditions were: overall survival or disease free survival, hazards radio (HR), and 95% confidence interval.

### 2.2. Analysis of IRAK1 Expression in Hepatocellular Carcinoma and Its Associations with Clinicopathological Parameters

UALCAN (http://ualcan.path.uab.edu/, accessed on 8 September 2021) [23,24] was used to analyze the expression of IRAK1 in hepatocellular carcinoma and its variation with clinicopathological parameters. The ‘TCGA gene analysis’ function was used and the dataset of LIHC was selected. Differences in the expression levels between the two groups were evaluated by *t* test. Further, LinkedOmics (http://linkedomics.org, accessed on 6 April 2022) [25] was used to explore variations in IRAK1 expression and overall survival of hepatocellular carcinoma with pathology TNM stages and radiation therapy status. The cohort LIHC was selected. The search datasets for IRAK1 expression and overall survival were ‘RNAseq’ and ‘clinical’, respectively. The target datasets for both were ‘clinical’. The statistical method was non-parametric test.

### 2.3. Immunohistochemistry Experiments on Liver Tissues from Clinical Specimens

Cancer tissue specimens were collected from 40 patients with hepatocellular carcinoma who received surgical treatment in the First Affiliated Hospital of Henan University of Science and Technology from 1 January 2010 to 1 March 2011. The cases included 29 males and 11 females, aged 32–73 years with an average age of 56 years. The tumor size was <5 cm in 18 cases, and ≥5 cm in 22 cases. The tumor grades comprised well differentiation (8 cases), moderate differentiation (16 cases), and moderate–poor or poor differentiation (16 cases). Para-carcinoma tissue specimens randomly selected from 10 patients were used as the control group. All the cases met the following criteria: being confirmed as hepatocellular carcinoma by histopathology; no malignant tumor of other organs; no immune system disease; no chemotherapy, radiotherapy, or other anti-tumor treatments before surgery; with clinical information including gender, age, tumor size, and tumor grade. The study was approved by the Medical Ethics Committee of the First Affiliated Hospital of Henan University of Science and Technology (approval date: 8 March 2021) and was conducted in accordance with the Declaration of Helsinki.

IRAK1 expression in hepatocellular carcinoma and para-carcinoma tissues was detected using the SP method. Anti-IRAK1 rabbit anti-human antibody, SP (rabbit IgG)-POD Kit and Mayer’s hematoxylin solution were purchased from Beijing Solarbio Science & Technology Co., Ltd. (Beijing, China). Paraffin block tissue specimens were continuously sectioned at 3 μm and baked on slides. The sections were dewaxed, hydrated, processed with 3% H_2_O_2_ solution for inactivation of endogenous enzymes, and immersed in citrate buffer solution (pH 6.0) under microwaves for antigen retrieval. Goat serum was used for blocking. The primary antibody (anti-IRAK1 antibody, diluted at 1:50) was added for incubation overnight at 4 °C. Afterwards, the secondary antibody (Bio-goat anti-rabbit IgG, diluted at 1:100) was added for incubation for 1 h at room temperature. Streptavidin-POD, 3,3′-diaminobenzidine (DAB), and hematoxylin were used for blocking, staining, and counterstaining, respectively. The positive control employed known-positive tissue sections. The negative control used phosphate buffered saline (PBS) instead of the primary antibody.

The staining area and intensity of tissue sections were semi-quantitatively analyzed, considering brown granules presented in the cytoplasm and nucleus as positive expression. In each section, five high magnification fields (×200) were randomly selected and analyzed by the ImageJ software (version 1.51) [26] to afford the percentage of positive area. The staining area was scored as 0 (<5%), 1 (≥5%~25%), 2 (≥25%~50%), 3 (≥50%~75%), and 4 (≥75%). The staining intensity was scored as 0 (no staining), 1 (weak, pale yellow), 2 (moderate, brownish yellow), and 3 (strong, brown). The overall score was calculated as ‘staining area × intensity’, with <3 as negative (−), ≥3~6 as weakly positive (+), ≥6~9 as moderately positive (++), and ≥9~12 as strongly positive (+++). The (+), (++), and (+++) were all positive expression.

The SPSS Statistics software (version 19, IBM, Armonk, NY, USA) was used for statistics. The χ^2^ test was performed, and *p* < 0.05 was considered statistically significant.

### 2.4. Molecular Docking Study

Fourteen representative natural compounds with anti-inflammatory and anti-tumor activities were selected from chemical classes of lignans, anthraquinones, phenanthraquinones, flavones, iridoid glycosides, triterpenoids, alkaloids, and phenols. They were schisandrin, rhein, cryptotanshinone, baicalin, luteolin, gentiopicroside, geniposide, oleanolic acid, cucurbitacin B, saikosaponin d, oxymatrine, matrine, harmine, and gingerol. They can be retrieved from PubChem (https://pubchem.ncbi.nlm.nih.gov/, accessed on 18 May 2022) [27] and ChemSpider (http://www.chemspider.com/, accessed on 19 May 2022) [28]. Drug likeness screening was performed at SwissADME (http://www.swissadme.ch/, accessed on 28 May 2022) [29] and confirmed at PubChem and ChemSpider. Compounds with any violation to Lipinski’s rule (molecular weight >500, Mlog*P* >4.15, hydrogen bond acceptors >10, or hydrogen bond donors >5) [30,31] were filtered out. Further, those with topological polar surface area (TPSA) > 140 Å^2^ were excluded [32,33]. The retained compounds were subjected to docking analysis.

The crystal structure of human IRAK1 (PDB code, 6BFN) [10] was downloaded from RCSB PDB (https://www.rcsb.org/, accessed on 4 May 2022) [34,35]. It is a co-crystal complex with an inhibitor ‘JH-I-25′ [10]. The protein structure was imported into PyMOL (version 2.4.0) [36] to remove solvent and ligand, and further processed with AutoDock Vina (version 1.1.2) [37] to add hydrogens and merge non-polar hydrogens. As for the small molecules, Open Babel (version 2.4.1) [38] was used to convert their chemical structures into mol2 files. The compounds were further processed for adding hydrogens and detecting torsion root. The grid box was adjusted to contain the active binding site with reference to the location of the original co-crystal ligand. Docking was performed in semi-flexible mode. Firstly, the original ligand JH-I-25 was re-docked into the active site successfully (RMSD < 1.5 Å, binding energy −10.2 kcal/mol), which verified the docking methodology. Selected natural compounds were then docked with the active site. For each docking, 20 binding modes were generated and ranked by binding affinity. The optimal mode was presented using PyMOL. Herein, JH-I-25, the original co-crystallized ligand and known inhibitor of IRAK1 [10], was used as a positive control for the molecular docking. The docking results of the natural compounds were compared with those of JH-I-25.

The entire in silico screening procedure is summarized in Scheme S1 (Supplementary Materials). Firstly, we selected 14 representative compounds from the main chemical classes of natural products. Secondly, we performed drug likeness screening based on Lipinski’s rule and the TPSA parameter. Thirdly, we performed molecular docking analysis, using re-docked original co-crystallized inhibitor as a positive control, to find out which compounds were able to bind into the inhibitory pocket with satisfactory binding energy. Finally, we considered those with both superior docking properties and very low TPSA values as the best hits.

## 3. Results

### 3.1. Results of Pan-Cancer, Gene Alteration, and Survival Analyses Regarding IRAK1

**Figure 1:**
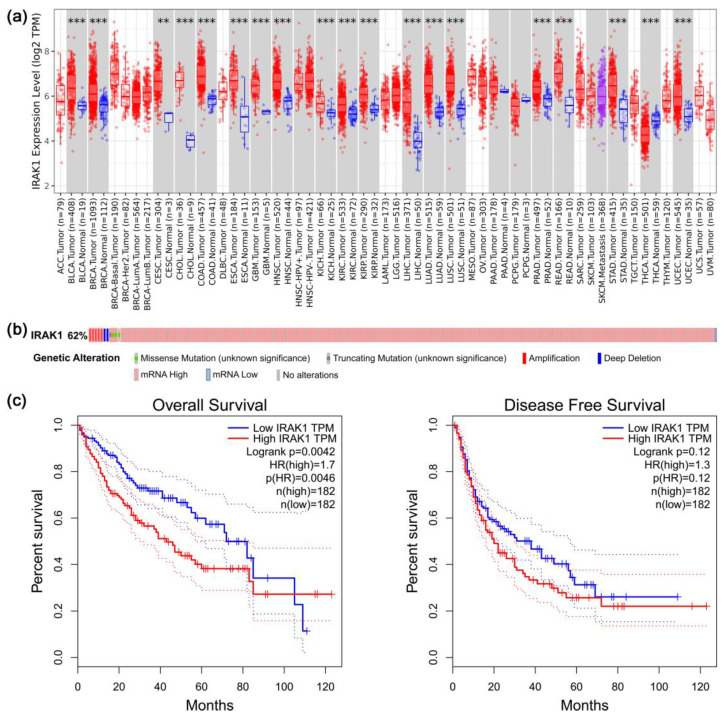
Pan-cancer, gene alteration, and survival analyses regarding interleukin-1 receptor-associated kinase 1 (IRAK1). (**a**) Pan-cancer analysis of IRAK1 expression. Comparisons between tumors and adjacent normal tissues are displayed in gray columns. The statistical significance is annotated as ** (*p* < 0.01), and *** (*p* < 0.001); (**b**) onco-print diagram for alterations of IRAK1 in hepatocellular carcinoma. The judgement of mRNA-high or -low was based on the expression *z*-scores calculated relative to normal samples and the set threshold (±2.0); (**c**) effects of IRAK1 expression on overall survival and disease-free survival of patients with hepatocellular carcinoma. The group cutoff was set at median, and, therefore, the high expression (samples with expression levels higher than this threshold) and low expression (samples with expression levels lower than this threshold) groups each accounted for 50% of the total LIHC cases.

There were 19 cancer types with significant differences in IRAK1 expression between tumor and adjacent normal tissues (*p* < 0.01; Figure 1a). Moreover, for most of them (18 cancer types, including hepatocellular carcinoma), IRAK1 expression was higher in tumors than in normal tissues (for hepatocellular carcinoma, the significance was *p* < 0.001). In contrast, there was only one cancer type, thyroid carcinoma (THCA), which showed lower expression of IRAK1 in tumor tissues.

Alterations of IRAK1 occurred in 217 out of 348 hepatocellular carcinoma cases, with a frequency of 62.4% (Figure 1b). Moreover, they were mostly mRNA-high (205 cases, 58.9%), relative to normal samples, in terms of expression *z*-scores and the set threshold (±2.0). By contrast, mRNA-low, relative to normal expression, only occurred in 1 case (0.3%). Other alteration types included mutation (1 case, 0.3%), deep deletion (2 cases, 0.6%), and multiple alterations (8 cases, 2.3%). As for the effects of IRAK1 expression on the survival of hepatocellular carcinoma patients, since the group cutoff (the expression threshold for splitting the high and low expression within the LIHC dataset) was set at median, the high expression (samples with expression level higher than this threshold) and low expression (samples with expression level lower than this threshold) groups each accounted for 50% of the total cases. Therefore, comparison was made between 182 cases of high expression and 182 cases of low expression (Figure 1c). The overall survival of the low expression group was prolonged in comparison with that of high expression group (*p* < 0.01). On the other hand, differential expression of IRAK1 had no significant effect on the disease-free survival (*p* > 0.05).

### 3.2. Expression Profiles of IRAK1 in Hepatocellular Carcinoma and Their Associations with Clinicopathological Parameters

**Figure 2:**
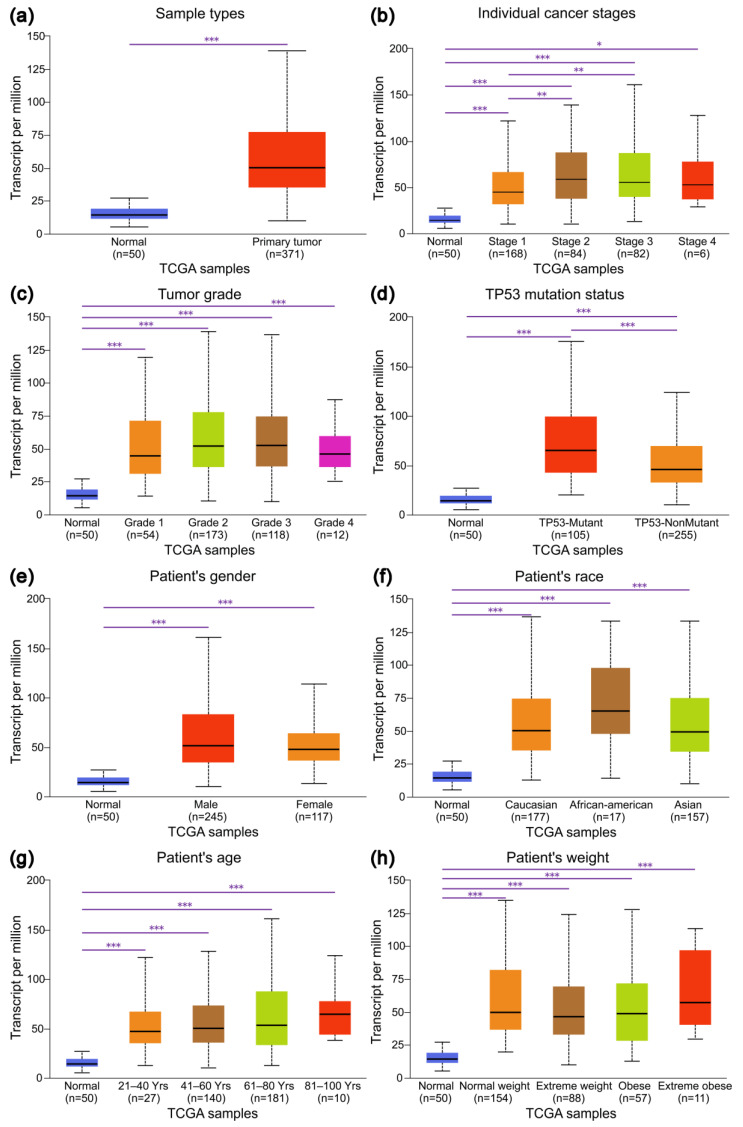
The expression levels of IRAK1 in hepatocellular carcinoma based on sample types and clinicopathological parameters. (**a**) Sample types; (**b**) individual cancer stages; (**c**) tumor grades; (**d**) TP53 mutation status; (**e**) gender; (**f**) race; (**g**) age; (**h**) weight. The statistical significance is annotated as * (*p* < 0.05), ** (*p* < 0.01), and *** (*p* < 0.001).

IRAK1 expressed higher in primary tumors than in normal tissues (*p* < 0.001; Figure 2a). Further, the expression in all pathological stages was higher than that in normal (*p* < 0.001 for normal vs. stages 1, 2, and 3; *p* < 0.05 for normal vs. stage 4; Figure 2b). Particularly, the expression in stages 2 and 3 was higher than that in stage 1 (*p* < 0.01). Moreover, IRAK1 was highly expressed in all tumor grades in comparison with normal (*p* < 0.001; Figure 2c). It was especially high in grades 3 and 2, although the difference between grades was not significant (*p* > 0.05). Importantly, the expression levels were significantly different between normal, tumor protein P53 (TP53) mutant, and TP53 non-mutant groups (all *p* < 0.001; Figure 2d). It was highest in tumors with TP53 mutant, which was followed by TP53 non-mutant, and lowest in normal tissues. In addition, there were significant differences between normal tissues and tumors of different genders/races/ages/weights (*p* < 0.001; Figure 2e–h); however, the differences within genders/races/ages/weights were not significant (*p* > 0.05).

**Figure 3:**
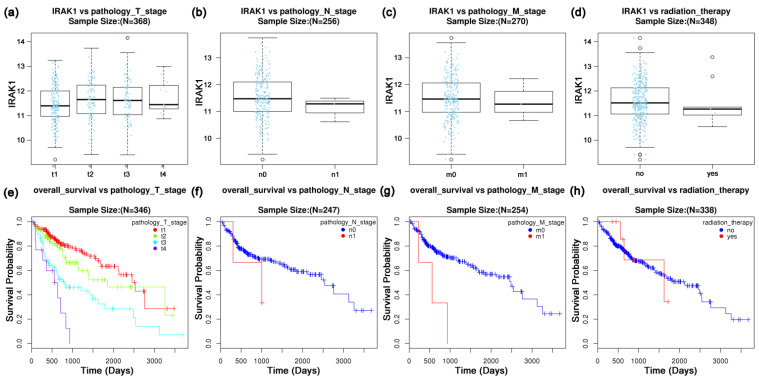
Variation of IRAK1 expression and overall survival of hepatocellular carcinoma based on pathology TNM stages and radiation therapy status. (**a**) IRAK1 vs. T stage (*p* = 0.1675, Kruskal-Wallis test); (**b**) IRAK1 vs. N stage (*p* = 0.2957, Wilcox test); (**c**) IRAK1 vs. M stage (*p* = 0.6619, Wilcox test); (**d**) IRAK1 vs. radiation therapy (*p* = 0.3961, Wilcox test); (**e**) overall survival vs. T stage (*p* = 0.1269); (**f**) overall survival vs. N stage (*p* = 0.2542); (**g**) overall survival vs. M stage (*p* = 0.0106); (**h**) overall survival vs. radiation therapy status (*p* = 0.9553). The statistics for panels (**e**–**h**) were Cox regression test.

Moreover, there seemed to be no significant variations in IRAK1 expression with pathology T, N and M stages, or radiation therapy status (all *p* > 0.05; Figure 3a–d). Nevertheless, the expression in pathology T2 and T3 stages was slightly higher than that in T1 stage. We further investigated the overall survival of hepatocellular carcinoma based on pathology TNM stages and radiation therapy. The survival rate of the M0 stage was significantly higher than that of the M1 stage (*p* < 0.05; Figure 3g). Moreover, the survival decreased gradually as pathology T stages proceeded from T1 to T4; nevertheless, without statistical significance (*p* > 0.05; Figure 3e). In addition, the overall survival was not significantly associated with N stage or radiation therapy status (both *p* > 0.05; Figure 3f,h).

### 3.3. IRAK1 Expression in Liver Tissues of Patients with Hepatocellular Carcinoma Experimentally Detected by Immunohistochemistry

**Figure 4:**
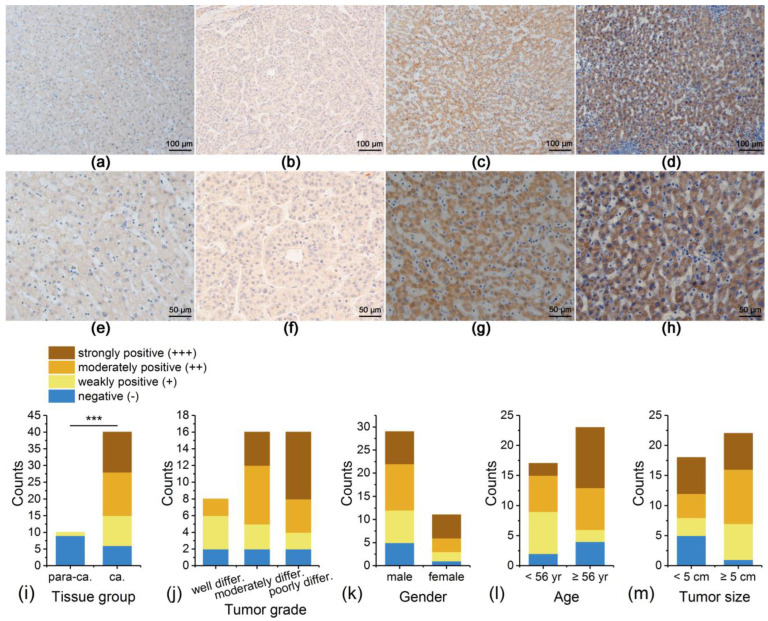
Typical immunohistochemistry staining for IRAK1 expression in the liver tissues from clinical specimens of patients with hepatocellular carcinoma. (**a**,**e**) Para-carcinoma tissue, negative (−); (**b**,**f**) well differentiated tumor tissue, weakly positive (+); (**c**,**g**) moderately differentiated tumor tissue, moderately positive (++); (**d**,**h**) poorly differentiated tumor tissue, strongly positive (+++). The magnifications for panels (**a**–**d**) are ‘×100′, and those for (**e**–**h**) are ‘×200′; (**i**–**m**) the distribution of counts of different staining in tissue groups, tumor grades, genders, ages, and tumor sizes. Statistical significance was evaluated based on the total positive rate, taking (+), (++), and (+++) into consideration. For tissue group, it was *p* < 0.001 (***). Others were not significant (*p* > 0.05).

The expression of IRAK1 was mainly located at the cytoplasm and nucleus, which showed pale yellow, brownish yellow, or brown staining granules under a microscope (Figure 4a–h). The positive expression was detected in 34 out of 40 cases of hepatocellular carcinoma tissues, accounting for 85%. As for para-carcinoma tissues, the positive expression was detected only in 1 out of 10 cases, accounting for 10%. Therefore, the positive rate of expression in hepatocellular carcinoma tissues was significantly higher than that in para-carcinoma tissues (χ^2^ = 18.006, *p* < 0.001; Figure 4i). Moreover, higher tumor grades (or poor differentiation) correlated with more strongly positive staining, although the total positive rates between grades showed no significant difference (*p* > 0.05; Figure 4j). There were no significant variations in total positive rates with gender, age, or tumor size (*p* > 0.05; Figure 4k–m). Nevertheless, a higher positive rate correlated slightly with larger tumor size (95.5% for size ≥5 cm; 72.2% for size <5 cm).

### 3.4. Docking Characteristics of IRAK1 with Selected Natural Compounds

Drug likeness screening of the 14 natural compounds filtered out five compounds (baicalin, geniposide, oleanolic acid, cucurbitacin B, and saikosaponin d) which were in violation of Lipinski’s rule or had too large TPSA values (>140 Å^2^) (Table S1, Supplementary Materials). The retained nine compounds (schisandrin, rhein, cryptotanshinone, luteolin, gentiopicroside, oxymatrine, matrine, harmine, and gingerol) were analyzed by the molecular docking method.

**Figure 5:**
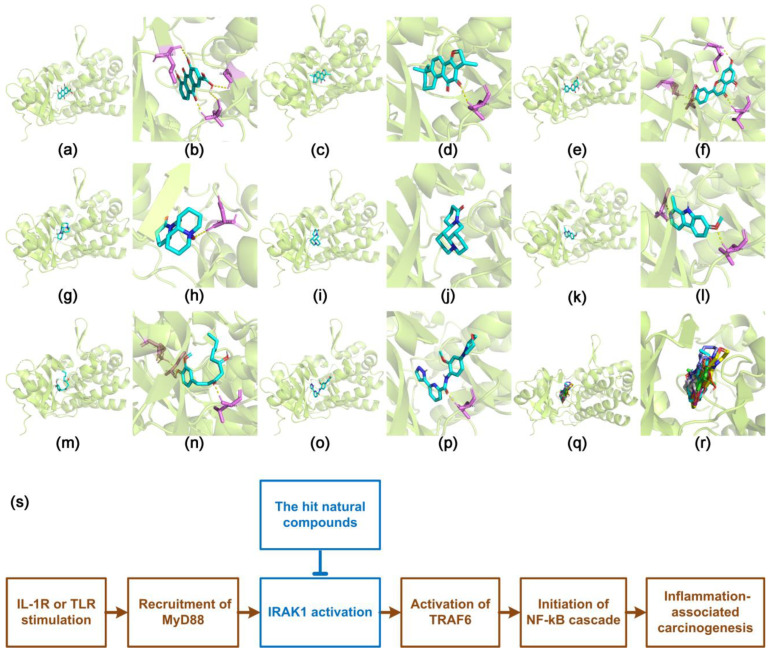
Docking poses of the selected natural compounds with IRAK1. (**a**,**b**) Rhein; (**c**,**d**) cryptotanshinone; (**e**,**f**) luteolin; (**g**,**h**) oxymatrine; (**i**,**j**) matrine; (**k**,**l**) harmine; (**m**,**n**) gingerol; (**o**,**p**) the re-docked original ligand inhibitor, JH-I-25; (**q**,**r**) superposed docking poses. Colors of the carbon skeleton: rhein in orange, cryptotanshinone in green, luteolin in violet, oxymatrine in blue, matrine in gray, harmine in magenta, gingerol in cyan, and JH-I-25 in yellow. Colors of other atoms: hydrogen (polar) in gray, nitrogen in blue, and oxygen in red; The panels (**a**,**c**,**e**,**g**,**i**,**k**,**m**,**o**,**q**) are in global view, whereas panels (**b**,**d**,**f**,**h**,**j**,**l**,**n**,**p**,**r**) are in active-site view; (**s**) proposed involvement of the hit compounds in the IRAK1/NF-κB signaling.

Further, seven compounds were successfully docked into the active site of IRAK1 (around LEU-291, ASP-358, and other residues), namely rhein, cryptotanshinone, luteolin, oxymatrine, matrine, harmine, and gingerol (Figure 5a–n). These molecules were well-accommodated into the inhibitory pocket. Moreover, their docking poses were well-superposed with each other, and with that of JH-I-25; the re-docked original co-crystallized ligand and known inhibitor of IRAK1 were used herein as positive control (Figure 5o–r). Their binding energy (−10.3 to −7.6 kcal/mol) was fundamentally comparable to that of JH-I-25 (−10.2 kcal/mol) (Table S2). Particularly, rhein, luteolin, cryptotanshinone, matrine, and harmine were most successfully docked, with binding energies of −10.3, −10.1, −9.4, −9.3, and −9.2 kcal/mol, respectively. Importantly, among them, matrine, harmine, and cryptotanshinone showed very low TPSA values (23.6, 37.9, and 43.4 Å^2^, respectively; Table S1). However, schisandrin and gentiopicroside were unable to be docked into the inhibitory pocket. None of the 20 generated binding modes for these two compounds were acceptable (Figure S1). Further, the possible involvement of the hit compounds in the IRAK1/NF-κB pathway was proposed (Figure 5s).

## 4. Discussion

IRAK1 is associated with inflammatory diseases and several types of cancers. It is overexpressed in most hematologic malignancies [11], non–small cell lung carcinoma [12], and hepatocellular carcinoma [13]. In breast cancer, its expression decreases after neoadjuvant chemotherapy, which is associated with a reduction in tumor size [14]. The knockdown of IRAK1 attenuates the growth of hepatocellular carcinoma cells [15]. Moreover, the inhibition of IRAK1 reduces the resistance of tumor cells against radiotherapy [16] and chemotherapy [17]. It is therefore expected that IRAK1 has the potential to be a prognosis indicator and therapeutic target for these diseases.

In this study, we systematically explored the significance of IRAK1 in the diagnosis, prognosis, and targeted therapy of hepatocellular carcinoma. We found that IRAK1 was highly expressed in most cancer types (including hepatocellular carcinoma) and may be a pan-cancer biomarker. As for hepatocellular carcinoma, the total alteration rate of IRAK1 was rather high, in which mRNA high relative to normal samples predominated. In combination with our further results that higher expression of IRAK1 was associated with shorter overall survival and disease-free survival (although for disease-free survival there was only a slight trend without statistical significance), it was proposed that IRAK1 could be a significant biomarker for the prognosis of hepatocellular carcinoma.

Our further analysis revealed specific associations between IRAK1 expression profiles and clinicopathological parameters of hepatocellular carcinoma. The results indicated a higher expression of IRAK1 in hepatocellular carcinoma tissues. Besides, IRAK1 correlated positively with pathology stages and tumor grades (for grades there was a slight trend without statistical significance). These were further confirmed by the immunohistochemistry results that the carcinoma tissues presented a higher positive expression rate of IRAK1 than para-carcinoma tissues. Besides, even the total rate of positive staining between tumor grades presented no significant difference, a higher grade correlated with more strongly positive staining. The immunohistochemistry also indicated a slight correlation between higher positive expression and larger tumor size. Ye et al. [13] and Yang et al. [14] reported significant correlations between IRAK1 expression and tumor size in hepatocellular carcinoma tissues and during neoadjuvant chemotherapy of breast cancer. Interestingly, we found that the expression of IRAK1 correlated positively with TP53 mutation. The TP53 gene is one of the most frequently mutated tumor suppressor genes in human cancer. Its mutation is generally associated with carcinogenesis and poor prognosis of patients with cancer. However, drugs targeting TP53 are not yet available [39]. Herein, our results provided a possible strategy for targeting TP53 via IRAK1. On the other hand, even though no significant associations were observed between IRAK1 and TNM stages or radiation therapy, the expression in the T2 and T3 stages was slightly higher than that in the T1 stage. Besides, the LIHC cases of stage N1, stage M1, and radiation therapy were all very few in number; therefore, possible associations with these parameters needs to be further explored. These findings suggested that the inhibition of IRAK1 could be a potential therapeutic strategy for hepatocellular carcinoma.

IRAK1 is a key node of the IRAK1/TRAF6/NF-κB signaling which is related to inflammation-associated carcinogenesis [10,11]. Upon the stimulation of IL-1R or TLR, the adaptor protein MyD88 (myeloid differentiation primary response protein 88) is recruited, which further recruits IRAK4 and IRAK1 to form a complex that further activates IRAK1. Consequently, IRAK1 in its active form interacts with TRAF6 and initiates the NF-κB cascade [10,11]. There are reports on synthetic IRAK1 inhibitors designed for therapeutic purposes against tumor or inflammatory diseases, such as JH-I-25 [10], oxfendazole (repurposed) [16], and others [11]. On the other hand, natural compounds with chemical diversity are a promising source for the development of anticancer agents [3]. However, there are few studies on the screening of IRAK1 inhibitors from natural compounds for targeted therapy of hepatocellular carcinoma. In a study, geranylgeraniol inhibited NF-κB activation in human macrophage-like cells via suppressing the expression of IRAK1 and TRAF6 [40].

Herein, we selected 14 representative natural compounds with anti-inflammatory and anti-tumor activities from the main chemical classes of bioactive natural products. The molecular docking afforded seven compounds with successful docking. Particularly, rhein, luteolin, cryptotanshinone, matrine, and harmine showed superior docking characteristics. More importantly, matrine, harmine, and cryptotanshinone showed very low TPSA values (<45 Å^2^), which indicated that they might have good cell membrane permeability. Therefore, cryptotanshinone, matrine, and harmine were considered as the best hit compounds with the potential to inhibit IRAK1 via binding to the inhibitory pocket. In contrast, schisandrin and gentiopicroside were unable to be docked to the active site, partially because they were too bulky in molecular volume to be accommodated within the inhibitory pocket. This implied that they might not act through interactions with IRAK1. On the other hand, baicalin, geniposide, oleanolic acid, cucurbitacin B, and saikosaponin d failed the drug likeness screening due to violation of Lipinski’s rule or too-large TPSA values. Lipinski’s rule is one of the most influential criteria for predicting the membrane permeability and bioavailability of small molecule drugs. Violations of the rule generally correlate with poor absorption or permeation [30,31]. TPSA is a key parameter that correlates with membrane permeability. Molecules with TPSA over 140 Å^2^ are poor in permeation through cell membranes [32,33]. Therefore, the above five compounds were not considered for molecular docking.

As for the selected natural compounds, rhein controls cancer cells via regulating pathways related to NF-κB, extracellular signal-regulated kinase (ERK), and phosphatidylinositol 3-kinase (PI3K) [41]. Luteolin has anti-inflammatory effects via interactions with Janus kinase (JAK)/STAT3 and NF-κB pathways, and presents anti-cancer activities via modulating glucose metabolism, cell growth, and apoptosis [42]. Cryptotanshinone induces apoptosis and inhibits proliferation via STAT3-related pathways, and inhibits inflammation via the NF-κB pathway. Interestingly, it reverses drug resistance in tumors [43]. Harmine suppresses collagen production in hepatic stellate cells by inhibiting dual-specificity tyrosine-regulated kinase 1B (DYRK1B) [44]. Oxymatrine and matrine inhibit cell proliferation, induce cell cycle arrest, promote apoptosis, and restrain angiogenesis [45]. Gingerol modulates several cancer-related pathways, including NF-κB, STAT3, and activator protein-1 (AP-1) [46].

We herein proposed the possible involvement of the hit compounds in the IRAK1/NF-κB pathway (Figure 5s). They might inhibit IRAK1 via binding to the inhibitory pocket, and consequently suppress the IRAK1/NF-κB signaling, so as to prevent the inflammation-associated carcinogenesis. Our findings revealed, for the first time, the potential of the hit natural compounds as IRAK1 inhibitors and IRAK1/NF-κB signaling suppressors to be used in targeted therapy for hepatocellular carcinoma. These compounds may be also used in combination with currently available systemic drugs for better treatment of hepatocellular carcinoma. On the other hand, IRAK1 is not the sole protein involved in the pro-inflammatory immune response. Specific mechanisms of how the hit compounds may affect the interactions of IRAK1 with other proteins in the signaling are still to be investigated in further studies.

## Data Availability

The data presented in this study are contained in the article and in the supplementary materials.

## Supplementary Materials

The following supporting information can be downloaded at: https://www.mdpi.com/article/10.3390/curroncol29110700/s1. Scheme S1: The entire in silico screening procedure of the molecular docking analysis; Figure S1: Docking poses of schisandrin and gentiopicroside which failed the molecular docking for they were unable to be docked into the inhibitory pocket; Table S1: Chemical information and drug likeness screening of the 14 representative natural compounds with anti-inflammatory and anti-tumor activities; Table S2: Molecular docking results of the selected natural compounds after drug likeness screening and the original co-crystallized inhibitor with IRAK1.
