# Supplementary material for: Expression of IRAK1 in Hepatocellular Carcinoma, Its Clinical Significance, and Docking Characteristics with Selected Natural Compounds
Source: Curr Oncol. 2022 Nov 18;29(11):8904–16. doi: 10.3390/curroncol29110700 (PMC9689133; doi:10.3390/curroncol29110700)
Supplement: Supplementary file 1 [file curroncol-29-00700-s001.zip › curroncol-2003032-supplementary.pdf]

Supplementary Materials

Expression of IRAK1 in hepatocellular carcinoma, its clinical significance, and docking characteristics with selected natural compounds

Chaoying Song, Xinyu Gu and Ruifang Li*

Department of Pharmaceutical Sciences, School of Basic Medical Sciences, Henan University of Science and Technology, 471023, Luoyang, China

* Correspondence: ylliruifang@163.com; Tel: + 86 150 9019 5676

Table legends

Scheme S1. The entire in silico screening procedure of the molecular docking analysis.

Figure S1. Docking poses of schisandrin and gentiopicroside which failed the molecular docking for they were unable to be docked into the inhibitory pocket.

Table S1. Chemical information and drug likeness screening of the 14 representative natural compounds with anti-inflammatory and anti-tumor activities.

Table S2. Molecular docking results of the selected natural compounds after drug likeness screening and the original co-crystallized inhibitor with IRAK1.

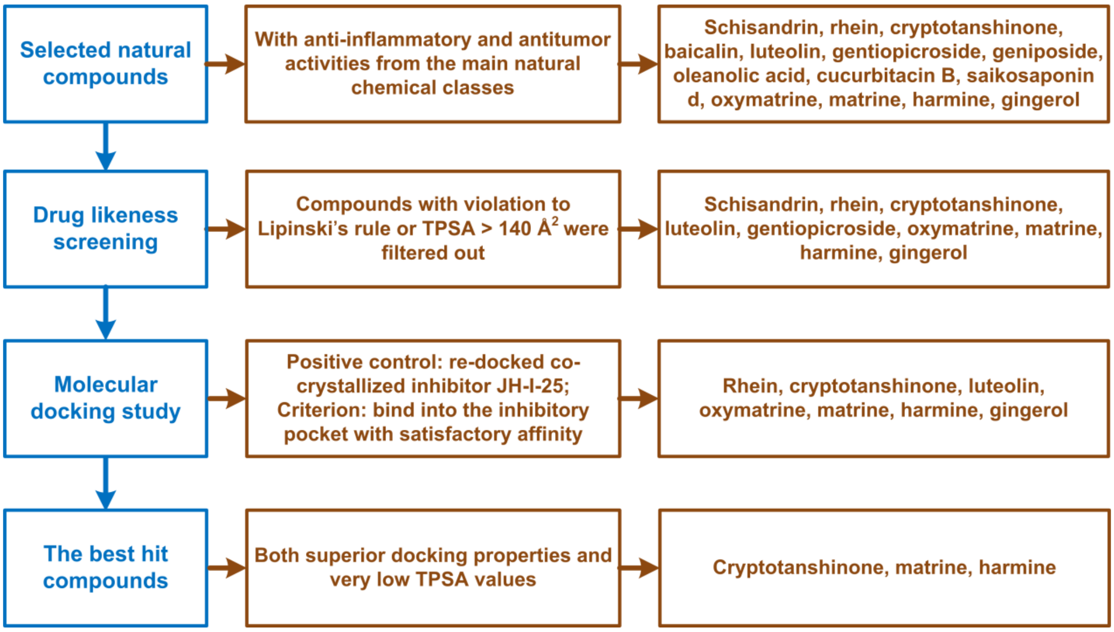

Scheme S1. The entire in silico screening procedure of the molecular docking analysis.

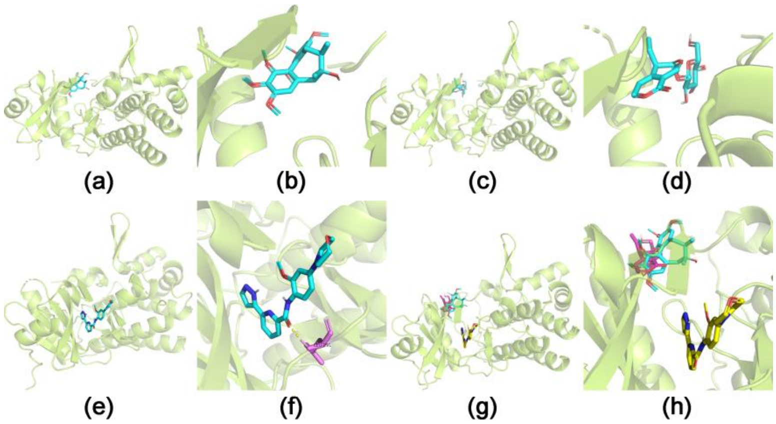

Figure S1. Docking poses of schisandrin and gentiopicroside which failed the molecular docking for they were unable to be docked into the inhibitory pocket. (**a, b**) Schisandrin; (**c, d**) Gentiopicroside; (**e, f**) The re-docked original ligand inhibitor, JH-I-25; (**g, h**) Superposed poses. Colors of the carbon skeleton: schisandrin in cyan, gentiopicroside in violet, JH-I-25 in yellow. Colors of other atoms: hydrogen (polar) in gray, nitrogen in blue, oxygen in red; The panels (a, c, e, g) are in global view, whereas panels (b, d, f, h) are in part view.

Table S1. Chemical information and drug likeness screening of the 14 representative natural compounds with anti-inflammatory and anti-tumor activities.

Category	Name	Chemical structure	Molecular formula	CAS NO.	PubChem CID	Biological activities	MW	Mlog*P*	H-A	H-D	Lipinski violations	TPSA, Å^2
Lignans	Schisandrin	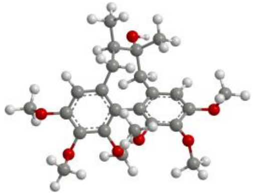	$C_{24}H_{32}O_7$	7432-28-2	23915	anti-inflammatory<br>protect against liver injury<br>anti-cancer	432.5	1.77	7	1	0	75.6
Anthraquinones	Rhein	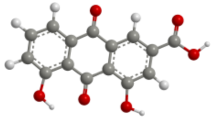	$C_{15}H_8O_6$	478-43-3	10168	hepatoprotective<br>anti-cancer<br>anti-inflammatory	284.2	0.29	6	3	0	111.9
Phenanthraquinones	Cryptotanshinone	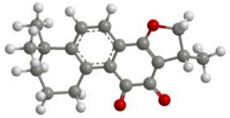	$C_{19}H_{20}O_3$	35825-57-1	160254	anti-tumor<br>anti-inflammatory	296.4	2.36	3	0	0	43.4
Flavones	Baicalin	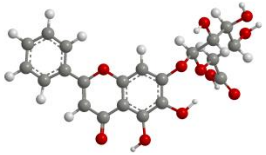	$C_{21}H_{18}O_{11}$	21967-41-9	64982	anti-inflammatory<br>antineoplastic<br>anticoronaviral<br>antibacterial	446.4	−1.63	11	6	2<br>H-A > 10,<br>H-D > 5	183.0
Flavones	Luteolin	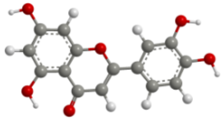	$C_{15}H_{10}O_6$	491-70-3	5280445	anti-inflammatory<br>immune system modulatory<br>antineoplastic	286.2	−0.03	6	4	0	107.0

Iridoid glycosides	Gentiopicroside	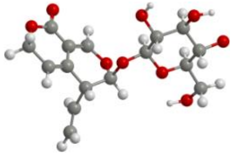	$C_{16}H_{20}O_9$	20831-76-9	88708	hepatoprotective anti-inflammatory	356.3	−1.42	9	4	0	134.9
Iridoid glycosides	Geniposide	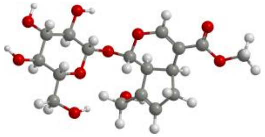	$C_{17}H_{24}O_{10}$	24512-63-8	107848	anti-inflammatory antioxidative antiproliferative	388.4	−1.86	10	5	0	155.1
Triterpenoids	Oleanolic acid	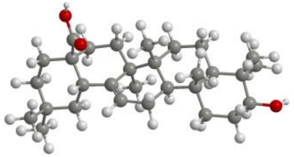	$C_{30}H_{48}O_3$	508-02-1	10494	antitumor hepatoprotective protect against liver injury	456.7	5.82	3	2	1 Mlog*P* > 4.15	57.5
Triterpenoids	Cucurbitacin B	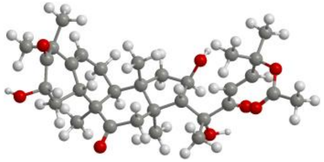	$C_{32}H_{46}O_8$	6199-67-3	5281316	antitumor inhibit proliferation anti-inflammatory	558.7	1.76	8	3	1 MW > 500	138.2
Triterpenoids	Saikosaponin d	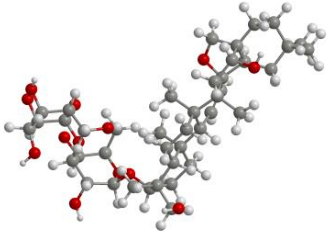	$C_{42}H_{68}O_{13}$	20874-52-6	107793	antitumor anti-inflammatory	781.0	0.18	13	8	3 MW > 500, H-A > 10, H-D > 5	208.0
Alkaloids	Oxymatrine	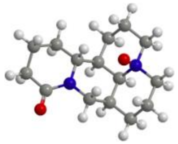	$C_{15}H_{24}N_2O_2$	16837-52-8	114850	anti-cancer anti-inflammation protect against liver injury	264.4	0.39	2	0	0	38.4

Alkaloids	Matrine	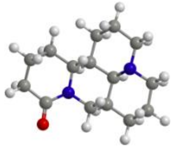	$C_{15}H_{24}N_2O$	519-02-8	91466	anti-inflammation alleviate liver injury anti-cancer	248.4	2.27	2	0	0	23.6
Alkaloids	Harmine	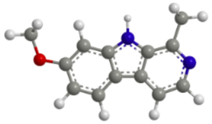	$C_{13}H_{12}N_2O$	442-51-3	5280953	anti-cancer anti-inflammatory	212.3	1.56	2	1	0	37.9
Phenols	Gingerol	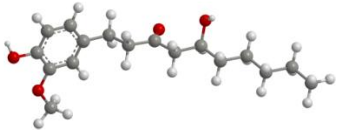	$C_{17}H_{26}O_4$	23513-14-6	442793	anti-inflammatory anti-cancer protective against toxins	294.4	2.14	4	2	0	66.8

Notes: MW, molecular weight; H-A, hydrogen bond acceptors; H-D, hydrogen bond donors; TPSA, topological polar surface area.

Table S2. Molecular docking results of the selected natural compounds after drug likeness screening and the original co-crystallized inhibitor with IRAK1.

Compounds	Outcome of the docking	Binding energy (kcal/mol)	Residues of hydrogen bonding interactions
Schisandrin	Unable to be docked to the active site	Not applicable	Not applicable
Rhein	Docked to the active site successfully	−10.3	LEU-291, ILE-218, GLY-294
Cryptotanshinone	Docked to the active site successfully	−9.4	LEU-291
Luteolin	Docked to the active site successfully	−10.1	LEU-291, ASP-358, LYS-239, ILE-218
Gentiopicroside	Unable to be docked to the active site	Not applicable	Not applicable
Oxymatrine	Docked to the active site successfully	−8.4	SER-344
Matrine	Docked to the active site successfully	−9.3	None
Harmine	Docked to the active site successfully	−9.2	LEU-291, ASP-358
Gingerol	Docked to the active site successfully	−7.6	LEU-291, ASP-358, LYS-239
Original ligand inhibitor JH-I-25	Docked to the active site successfully	−10.2	LEU-291
